# The migrating mediators and the interaction associated with the use of essential public health services: a cross-sectional study in Chinese older migrants

**DOI:** 10.1186/s12877-020-01878-0

**Published:** 2020-11-16

**Authors:** Chengxu Long, Shangfeng Tang, Ruoxi Wang, Lu Ji, Yang Wang, Tailai Wu, Zhifei Li, Zhanchun Feng

**Affiliations:** 1 School of Medicine and Health Management, Tongji Medical College, Huazhong University of Science and Technology, 13 Hangkong Road, Wuhan, 430030 Hubei China; 2 China National Center for Biotechnology Development, 16 West Sihuan Middle Road, Beijing, 100039 China

**Keywords:** Essential public health services, Health services utilization, Older migrants, China

## Abstract

**Background:**

Despite the incremental implementation of the essential public health services (EPHS) during the last decade, the goal of EPHS’s equalization is impossible to cannot be achieved without appropriate policies targeting older migrants. Therefore, this study aims to examine whether the supply side meets the needs of older migrants and to explore the relationships among health status, the use of health services, and diverse factors.

**Methods:**

The data were derived from a national cross-sectional dataset (*N* = 11,161) of the 2015 Chinese Migrant Dynamic Monitoring Survey. Mediating effects analysis and moderating effects analysis were conducted to explore the interactions between physical status and the use of EPHS in older migrants such as physical examination, health record, and follow-up services.

**Results:**

The use of physical examination, health record, and follow-up services were correlated with each other. Household income, migrating for employment, and migrating for offspring were negatively associated with the use of EPHS. A positive association was observed between the use of EPHS and willingness for long-stay. The mediating effects of household income, migrating for employment, migrating for offspring, and willingness for long-stay were observed on the relationship between physical status and the use of EPHS. The moderating effects of household income and migrating for employment were discovered.

**Conclusion:**

Public health policies that may be worthy of consideration include further enhancing the delivery capacity of primary health institutions, integrating professional clinical resources into the primary health system, and launching the target policies to improve the accessibility of EPHS in older migrants.

## Background

“Health for all” has gradually become the core effort to achieve the Sustainable Development Goal [1, 2]. The depth-released health needs have attracted more attention around the worldwide. An efficient delivery system and sufficient use of health services are the most effective approach to achieve population health [2]. In China, the equalization of public policies aims to ensure citizens with equal access to essential public health services (EPHS), regardless of gender, age, place of residence, or household income. However, the equalization of EPHS for migrants was one of the hardest nuts to crack. The use of EPHS was insufficient compared with the general populations, especially the older migrants’ health needs were generally ignored due to the complexity of the migration and the restrictive Hukou system.

Historically, the Chinese population management policy has been formed on the Hukou system, linked to different types of social security, such as employment, retirement, education, health insurance, et al. [3]. This unique culture and policy environment resulted in migrants being excluded from the social security system [4]. Simultaneously, urbanization and industrialization have accelerated the development process of the national economy and society. The older migrant, who used to be migrant workers, received more attention in the last decade. As forecasted, China’s aging rate might increase to 34.1% by 2050, which is much higher than the global average of 21.2% [5]. The number of older migrants aged over 60 in China reached 9.34 million, accounting for 7.2% of the total floating population [6].

Give the importance of this obstacle on the way of public services equalization, the work of Essential Public Services Equalization for Migrants was launched in 2013. “Health China 2030” strategy calls for integrating health into all policies, especially in protecting people’s health in all directions, entire life-cycle, and adhering to the goal of “health in all”. Compared with the studies on the use of health services among the general population, the issues on the older migrants await more exploration [7, 8]. Extant studies focused on the indirect effects of migrating and household factors on the relationships between physical status and the use of EPHS, especially for improving the equalization of EPHS among older migrants. Therefore, this study aims to examine whether the supply side meets the needs of older migrants, to explore the associated mediators and the moderators in the relationships between health status and the use of EPHS, and to provide implications for China and other developing countries that face similar challenges.

## Theoretical background and hypothesis development

### The use of essential public health services

Using EPHS is an effective way to enhance the social and economic benefits for the public, which illustrates whether the population’s primary health needs are timely satisfied or not. The package of EPHS for people aged over 65 years includes the health services provided in community institutions such as annual physical examinations (PE), health record (HR), follow-up services (FS) for patients with chronic disease [9, 10]. Actually, the use of PE, HR, and FS for chronic diseases are the recommended core indicators to assess the performance of the public health system in China [11].

### Physical status and household income

Health refers to the person’s status and integrity in three dimensions, namely physical, mental, and social aspects. Physical health is the core element, which directly indicates whether the body suffers from the illness. Evidence showed that those with poor physical status or high household income tended to receive more health services than their counterparts [4]. Those with worse physical status are more likely to be unemployed among migrants, then easily result in low economic status [12]. It is noteworthy that the mediating effect of household income has not been examined. Thus, this study hypothesized that monthly household income is a mediator between physical status and the use of EPHS for the older migrants. Specifically, an incremental number of health needs emerge as the deterioration of physical health [13]. Theoretically, one’s health needs could be converted to demand when healthcare services are provided at appropriate prices. Then, it might be satisfied by seeking health services. Since the older migrant’s family has low social-economic status, medical expenditure is a vital determinant in the use of health services. Due to subjective or objective factors such as limited economic capacity, people may not turn need into demand [14]. The mediating effect of household income might provide evidence for policymakers to adjust welfares policy for the older migrants. Therefore, this study proposed the following hypotheses:

**H1.** Older migrants with weaker physical status are more likely to utilize EPHS.

**H2.** Monthly household income has a mediating effect on the relationship between physical status and the use of EPHS.

### Population migration

To eliminate poverty, the government implemented strategies to promote industrialization and urbanization. As a result, millions of people migrated across geographical boundaries. Employment and migrating for offspring constituted to be the main reasons for migration [6]. The majority of older migrants who migrated for employment might have better health status and poor social-economic status, and their health demand seemed to be restrained [4]. Older migrants who migrated for offspring were likely to have better health status [4]. However, traditional Chinese parents always hold the obligation and priority in guardian roles, which may result in ignoring their health needs [8]. The findings shed light on the targeted policies towards the minorities migrated for employment or offspring. Therefore, limited health needs are less likely to be converted into demand or even the use of health services. Here are the following hypotheses:

**H3.** Migrating for employment has a mediating effect on the relationship between physical status and the use of EPHS.

**H4.** Migrating for offspring has a mediating effect on the relationship between physical status and the use of EPHS.

Willingness for long-stay directly refers to that older migrants have to or even already adapt to the new environment and challenges ahead. Older migrants with a willingness for long-stay have better physical status, while few studies examined its mediating effects. During the duration of long-stay, migrants are gradually integrated into the new community [15]. Additionally, with the extensions of the length of the migrating time, previous nostalgia gradually fades, and the social networks are reconstructed. They are more likely to adapt to the new lifestyles and change health behaviors [16]. Hence, those who wish to stay in the immigrating community are more likely to release health needs and seek healthcare proactively. The result may provide references for helping older migrants integrate into the immigrating community. As such, this study proposed the following hypotheses:

**H5.** Willingness for long-stay has a mediating effect on the relationship between physical status and the use of EPHS.

**Fig. 1:**
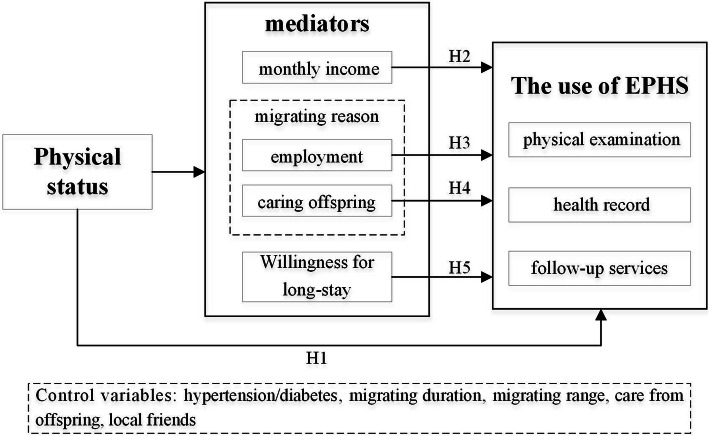
Hypothetical framework. EPHS: essential public health services

In conclusion, physical status directly influences the older migrants’ use of EPHS. Physical status has impacts on household income, migrating factors, and willing for long-stay family social support, which further influences the use of EPHS; that is, household income, migrating factors, and willing for long-stay may play mediating roles between physical status and the older migrants’ use of EPHS. Thus, this study proposed a hypothetical framework, as presented in Fig. 1.

## Methods

### Data

The data we used was a national cross-sectional dataset derived from the 2015 Chinese Migrant Dynamic Monitoring Survey (*N* = 11,161). The stratified, multi-stage, scale-oriented Probability Proportionate to Size method was employed in the sampling strategy. In total, approximately 10,000 sample points covered 32 provincial-level units, and annual reports of the migrants were collected in different districts. The self-reported questions were investigated anonymously without any deletion. Ethics approval was not applicable since it was a secondary analysis of a public dataset. The details of the procedures were available at the website of http://www.chinaldrk.org.cn/wjw/#/application/index as long as being a registered member.

### Variables

Physical examination and follow-up services are related to the control of hypertension and diabetes. As Table 1 shown, the use of EPHS was designed as the dependent variable, which was measured by one’s annual physical examination, follow-up services, and health record. The respective choices in the questionnaire were coded as Yes (1) and No (0). These three services are representatives of the EPHS package. Physical examination is the first and necessary step, and the follow-up services are related to hypertension and diabetes, which are one of the most prevalent chronic diseases in China [17]. All the results of the examination and treatment are stored in the health record. According to the items classified by the professional officers in the China Migrant Population Service Centre, the physical status of the older migrants was designed as the independent variable, which was categorized as cannot self-care (1), unhealthy but can self-care (2), generally healthy (3), and healthy (4).

**Table 1:** Variables and assignments

Variables	Assignments
*Dependent variable*	
Annually free physical examination	0 = no; 1 = yes
Follow-up services	0 = no; 1 = yes
Health record	0 = no; 1 = yes
*Independent variable*	
physical status	1 = cannot self-care; 2 = unhealthy but can self-care; 3 = generally healthy; 4 = healthy;
*Health*	
hypertension/diabetes	1 = no; 2 = yes
*Economic factors*	
Monthly household income after tax	1 = < ¥5000; 2 = ¥5000–10,000; 3= > ¥10,000
*Migration*	
migrating for employment	1 = no; 2 = yes
migrating for offspring	1 = no; 2 = yes
migrating duration	1 = < 5 years; 2 = 5–10 years; 3= > 10 years
migrating range	1 = cross-county;2 = cross-city; 3 = cross-provincial; 4 = cross-border
willingness for long-stay	1 = no; 2 = yes
*Family*	
receiving care from offspring	1 = no; 2 = yes
*Social support*	
numbers of local friends	1 = 0; 2 = 1–5; 3= > 5

Based on the hypotheses and variables’ availability in the database, the following variables were defined in the research to explore the effect of the mediators and moderators: health (hypertension/diabetes); economic factors (monthly household income after-tax); migration (migrating for employment, migrating for offspring, migrating duration, migrating range, and willingness for long-stay); family (receiving care from offspring); social support (numbers of local friends). Specifically, hypertension/diabetes refers to the participants suffered from one condition, either hypertension or diabetes. Migrating for offspring and migrating for employment are derived from the migrating reasons in the data set. Willingness for long-stay was defined as whether the older migrants wish to stay in the immigrating community in the long-term. The number of local friends indicated the social relations of older adults in the immigrating area.

### Analytical strategy

The analytical strategy was combined with correlation analysis, mediating effects analysis, and moderating effects analysis. Firstly, the correlations between the potential factors were conducted by correlation analysis. Then, mediating effect tests were conducted to explore the associations between the use of EPHS and underlying factors on the basis of the three-step method proposed by Baron [18]. The criteria for conducting the analysis of mediating effects were shown as follows: (1) the statistical significance was observed on the relationships between the independent variable and the dependent variable, so was the relationships between the independent variable and the mediator; (2) the link of the mediator in the regression model that contained independent variable and mediator was statistically significant [19].

Simultaneously, as proposed procedure [20], moderating effects analysis was performed through hierarchical regression analysis, which included the variables such as physical status, potential moderating variables, and the use of EPHS. The criteria for conducting the analysis of moderating effects were shown as follows: the link of the interaction term in the regression model contained independent variable, moderator, and interaction term was statistically significant. All the Statistics were performed by SPSS23.0. The *p* value less than 0.05 was regarded as significant. The *, ** and *** refers to *p* < 0.05, *p* < 0.01 and *p* < 0.001, respectively.

## Results

The coefficients of correlation were illustrated in Table 2. The use of PE, HR, and FS were positively correlated with each other. The variables of monthly household income and migrating for offspring were negatively correlated with the use of EPHS. However, physical status was correlated with the use of HR and FS, while it was positively correlated with the use of PE, which was partially confirmed the H1. Similarly, migrating for employment and willingness for long-stay merely were correlated with the use of PE and HR. However, it was not significantly correlated with the use of FS.

**Table 2:** Correlations of variables

	Variable	1	2	3	4	5	6	7	9	10	11	12	13	14
1	physical examination	–												
2	health record	0.340***	–											
3	follow-up service	0.429***	0.302***	–										
4	physical status	0.025**	−0.050***	− 0.046*	–									
5	hypertension/diabetes	0.022*	0.006	–	− 0.245***	–								
6	monthly household income	−0.050***	− 0.066***	− 0.035*	0.146	0.049***	–							
7	migrating for employment	−0.021*	− 0.041***	0.029	0.156***	−0.117***	− 0.158***	–						
8	migrating for offspring	−0.029**	− 0.039***	− 0.090***	0.125***	0.015	0.242***	−0.363***	–					
9	migrating duration	0.007	0.049***	−0.015	−0.047***	0.048***	−0.035***	0.043***	−0.052***	–				
10	migrating range	−0.128***	−0.124***	− 0.081***	0.109***	0.002	0.201***	0.020*	0.096***	0.090***	–			
11	willingness for long-stay	0.141***	0.303***	0.089	0.090*	−0.089*	−0.194***	0.006	0.148***	0.065	–	–		
12	care from offspring	−0.019	0.055**	0.026	0.085***	0.004	0.054*	0.005	0.010	− 0.034	− 0.053*	− 0.003	–	
13	local friends	0.140***	0.098***	0.094***	0.100***	−0.043***	−0.014	0.084***	−0.044***	0.099***	−0.068***	0.150***	0.040	–

* *p* < 0.05. ** *p* < 0.01. *** *p* < 0.001

### Test of mediating effects

As Table 3 illustrated, all of the mediating effects of variables were significant in the pathway from physical status to the use of PE, HR, FS (*N* = 11,161). The significant relations between the use of PE and physical status and migrating for employment were observed in the regression, whereas the coefficients of the relationship between physical status and the use of PE was smaller than that of the relationship between physical status and migrating for employment. Thus, migrating for employment partially mediated the effect of physical status on the use of PE (H3). Similar findings were observed in household income, migrating for offspring, migrating range, willingness for long-stay (H2, H4, and H5). The coefficient of physical status in the regression model that contained the variable of local friends was not significant, which meant that local friends fully mediated the effect of physical status on the use of PE.

**Table 3:** Results of mediating effects

IV	M	DV	IV-DV	IV-M	(IV + M)-DV	
					IV	M
Use of physical examination						
status	hypertension/diabetes	PE	0.035***	−0.253***	0.044***	0.033**
status	household income	PE	0.035***	0.144***	0.044***	−0.062***
status	migrating for employment	PE	0.035***	0.157***	0.040***	−0.028**
status	migrating for offspring	PE	0.035***	0.138***	0.040***	−0.034**
status	migrating range	PE	0.035***	0.109***	0.049***	−0.130***
status	willingness for long-stay	PE	0.035***	0.094*	0.099*	0.131**
status	local friends	PE	0.035***	0.119***	0.018	0.143***
Use of health record						
status	household income	HR	−0.044***	0.144***	−0.035***	−0.066***
status	migrating for employment	HR	−0.044***	0.157***	−0.039***	−0.035***
status	migrating for offspring	HR	−0.044***	0.138***	−0.039***	−0.034***
status	migrating duration	HR	−0.044***	−0.049***	− 0.042***	0.049***
status	migrating range	HR	−0.044***	0.109***	−0.032**	−0.115***
status	willingness for long-stay	HR	−0.044***	0.094*	0.076*	0.296***
status	care from offspring	HR	−0.044***	0.089***	−0.040	0.059**
status	local friend	HR	−0.044***	0.119***	−0.057***	0.107***
Use of follow-up services						
status	migrating for offspring	FS	−0.043*	0.138***	−0.023	−0.084***
status	migrating range	FS	−0.043*	0.109***	−0.032	−0.077***
status	local friend	FS	−0.043*	0.119***	−0.057**	0.103***

*IV* independent variable; *M* mediator; *DV* dependent variable; *PE* physical examination; *HR* health record; *FS* follow-up services

* *p* < 0.05. ** *p* < 0.01. *** *p* < 0.001

Similarly, migrating range, household income, migrating for employment, migrating for offspring, migrating duration, and willingness for long-stay partially mediated the effect of physical status on the HR (H2-H5). Receiving care from offspring fully mediated the effect of physical status on the health record. Besides, migrating for offspring and migrating range fully mediated the effect of physical status on the use of FS (H4), while the partially mediating effect of local friends was discovered in the relationship between physical status and the use of FS.

### Test of moderating effects

In terms of the use of PE, Model 1 and Model 2 were developed to estimate the associations of physical status and migrating for employment with their interaction on the use of PE. Containing the physical status and migrating for employment in the regression, the significant coefficient of the interaction term was observed in the model. Thus, migrating for employment moderated the relationship between physical status and the use of PE. Similarly, the migrating range moderated the relationship between physical status and HR, while monthly household income moderated the relationship between physical status and the use of FS. More details are shown in Table 4.

**Table 4:** Results of moderating effects

Independent variable	Physical Examination		Health Record		Follow-up services	
	M1	M2	M1	M2	M1	M2
physical status	0.033***	−0.142	−0.032**	0.032	−0.036	0.089
migrating for employment	−0.016	−0.063*				
migrating range			−0.115***	−0.003		
household income					−0.030	0.171*
Interaction						
physical status*migrating for employment		0.175*				
physical status*migrating range				−0.137**		
physical status*income						−0.265*
R^2^	0.002	0.002	0.015	0.016	0.003	0.005
F	8.413***	6.899***	85.035***	58.978***	3.538*	4.559**

* *p* < 0.05. ** *p* < 0.01. *** *p* < 0.001

Generally, the results of hypotheses testing are reported in Table 5. H4 was fully confirmed, while H1–3 and H5 were partially confirmed in this study.

**Table 5:** Results of hypotheses testing

Hypothesis	Results		
	PE	HR	FS
H1: Physical status → the use of EPHS (−)	X (+)	O	O
H2: Mediating effect of household income on the relationship between physical status and the use of EPHS	O	O	X
H3: Mediating effect of migrating for employment on the relationship between physical status and the use of EPHS	O	O	X
H4: Mediating effect of migrating for offspring on the relationship between physical status and the use of EPHS	O	O	O
H5: Mediating effect of willingness for long-stay on the relationship between physical status and the use of EPHS	O	O	X

*O* supported; *X* unsupported; *PE* physical examination; *HR* Health Record; *FS* follow-up services

## Discussion

This study investigated the associated factors with the use of EPHS in Chinese older migrants and estimated the mediators and moderators on the paths that translated physical status to the use of EPHS. According to EPHS’ national manual, the follow-up services such as monitoring blood pressure or blood glucose should be covered no less than four times a year. Relevant examination results should be timely recorded in the electronic health record after each follow-up services. Unfortunately, this study did not find that the indirect effects of household income, migrating for employment, and willingness for long-stay on the use of FS. It might be explained by the rigid demand with small elasticity comparatively, which varied across different types of medical services [21]. Generally, follow-up services were mainly covered by older adults with chronic disease. Its sensitivity might decline with long-term implementations in older patients’ subjective proactiveness or objective conditions.

The use of self-reported data would lead to underestimated prevalence estimates [22]. The EPHS conducted by the primary care providers could actively detect the prevalence of relevant diseases. Evidence revealed that follow-ups with blood pressure control helped reduce the mortality of congestive heart failure [23, 24]. The EPHS is free for all, and it is originally designed to ensure the population in need with equal access to the related services. However, only 33.8% of older migrants used the PS. Further improvement on the coverage is needed in comparison to the general population (43.3%). Simultaneously, the FS coverage rate gap between the older migrants and those aged over 15 years reached 36.7% (34.6% Vs. 71.3%) [25]. This gap might be explained by the instability of migration, weak health literacy, inadequate publicity, or excessive worries about costly treatment expenses [26, 27]. In terms of household income, we found a moderating effect rather than a mediating effect in the relationship between physical status and the use of FS. The key point is that health status was weakly associated with family income, and some of the older migrants were retired with an unvaried pension. Besides, household income has an impact on the use of FS, which is related to further treatment costs [4]. Those with a better social-economic level are more likely to suffer from hypertension or diabetes, and they were more likely to seek follow-up services [28].

As we hypothesized, migrating for children or employment mediated the relationship between physical status and the use of EPHS. Previous research evidenced that those migrated for offspring or employment have better health status, resulting in a negative association with the use of healthcare [4]. However, it is noteworthy that migration leads to the reconstruction of family structure and intergenerational relationships [29, 30]. Meanwhile, family conflicts and existed family devotion might undermine the utilization of health services in older adults. Those who migrated for employment or offspring have to support the whole family rather than merely living for retirement. Besides, the fully mediating effects of migrating for children in the relationship between physical status and the use of FS might be explained by the gap between the supply side and demand side [31, 32]. EPHS are mainly provided by health community centers and its subordinate clinics in the urban areas, while chronic disease treatment is separately provided by secondary or tertiary hospitals [33]. Hence, patients with chronic disease prefer to obtain treatment from professional physicians rather than health workers who confront a severe confidence crisis.

Notably, the effect of migrating for employment on the use of EPHS should not be ignored. This study found that those who migrated for employment were less likely to use the services. It might be explained by the fact the better physical health status older adults have, the more work they did, no matter to reduce their family’s economic burden or prefer to take family responsibilities [34, 35]. Unfortunately, the Hukou system excluded the migrating workers and incurred discrimination in employment, pension, and healthcare [27, 36]. Interestingly, given the mediating effects of willingness for long-stay, the likelihood of the decision-making on establishing a health record was increased. With the extension of migration duration, older migrants could selectively integrate themselves into the new circumstances and obtain equal opportunities to the corresponding social benefits [16].

In the short run, the community is suggested to provide support for the older migrants to incorporate them into the new circumstances [37]. On-site consultation regarding EPHS might be an efficient way to improve older migrants’ health behaviors, especially for those who suffer from chronic diseases [38]. Simultaneously, the offspring are suggested to pay attention to the senior’s health needs. Meanwhile, older adults are encouraged to not regard seeking EPHS as a burden [39]. The care delivery is worthy of strengthening the integration of medical services and preventive service, enhancing the delivery capacity of community health service centers, and implementing the equalization of EPHS [40]. The policies that may be worthy of consideration include developing a comprehensive reform to promote equity in terms of employment, pension, and healthcare for the older migrants [26], which would help achieve the goal of the equalization of EPHS, enhance the intergenerational relationship and social stability, promote the urbanization, and response to the healthy aging.

### Limitation

Several limitations should not be ignored in this study. The last survey on older migrants was conducted in 2015, and the cross-sectional data could not provide implications for the long-term practice. Hence, further studies need to be conducted to confirm the findings and explore the latest associations for older migrants in China. This study focused on the existing variables in the data set, and other variables (intergeneration conflicts, living arrangements, social relations) should be further explored in the subsequent study.

## Conclusion

As the findings indicated, the use of EPHS (PE, HCR, and FS) was correlated with each other. Income, migrating for employment, migrating for offspring were negatively associated with the use of EPHS, while a positive association was observed in the relationship between willingness for long-stay and the use of EPHS. The mediating effects of household income, migrating for employment, migrating for offspring, and willingness for long-stay were observed on the relationship between physical status and the use of EPHS, while household income and migrating for employment demonstrated moderating effects in these relationships. Hence, policies that may be worthy of consideration include further developing the health system reform to promote the delivery capacity of primary health institutions, integrating the professional physicians into public health departments, and launching equality policies.

## Data Availability

The data that support the findings of this study are available from the 2015 China Migrant Dynamic Monitoring Survey, but restrictions apply to the availability of these data, which were used under license for the current study, and so are not publicly available. Data are available from the authors upon reasonable request and with permission of the Migrant Population Service Center of the Chinese National Health Commission on the website of http://www.chinaldrk.org.cn/wjw/#/application/index.

## Abbreviations

EPHS: Essential public health services
PE: Physical examination
HR: Health record
FS: Follow-up services

## References

[1] Haslegrave M (2013). Ensuring the inclusion of sexual and reproductive health and rights under a sustainable development goal on health in the post-2015 human rights framework for development. Reprod Health Matters.

[2] Bloom BS, Fendrick AM (1996). The tension between cost containment and the underutilization of effective health services. Int J Technol Assess Health Care.

[3] Shi L (1994). Elderly support in rural and suburban villages: implications for future support system in China. Soc Sci Med (1982).

[4] Zhang X, Yu B, He T, Wang P (2018). Status and determinants of health services utilization among elderly migrants in China. Global Health Res Pol.

[5] Grigoryeva IVL, Dmitrieva A, Sergeyeva O (2019). An aging population in the modern world: between work, education and health. In Switzerland.

[6] China NHCo (2016). China floating population development report 2015. In. Beijing.

[7] Meng Q, Fang H, Liu X, Yuan B, Xu J (2015). Consolidating the social health insurance schemes in China: towards an equitable and efficient health system. Lancet.

[8] Long C, Wang R, Feng D, Ji L, Feng Z, Tang S (2020). Social Support and Health Services Use in People Aged over 65 Years Migrating within China: A Cross-Sectional Study. Int J Environ Res Public Health.

[9] Tang S, Bishwajit G, Ji L, Feng D, Fang H, Fu H, Shao T, Shao P, Liu C, Feng Z (2016). Improving the Blood Pressure Control With the ProActive Attitude of Hypertensive Patients Seeking Follow-up Services Evidence From China. Med.

[10] Sun X, Chen Y, Tong X, Feng Z, Wei L, Zhou D, Tian M, Lv B, Feng D. The use of annual physical examinations among the elderly in rural China: a cross-sectional study. BMC Health Serv Res. 2014;14. 10.1186/1472-6963-14-16 PMC3925351 24423046

[11] Jing Z, Wang Y, Ding L, Tang X, Feng Y, Zhou C (2019). Effect of social integration on the establishment of health records among elderly migrants in China: a nationwide cross-sectional study. BMJ Open.

[12] Tang S, Long C, Wang R, Liu Q, Feng D, Feng Z (2020). Improving the utilization of essential public health services by Chinese elderly migrants: strategies and policy implication. J Glob Health.

[13] Parslow R, Jorm A, Christensen H, Jacomb P, Rodgers B (2004). Gender differences in factors affecting use of health services: an analysis of a community study of middle-aged and older Australians. Soc Sci Med.

[14] Hajek A, Koenig H-H (2019). Meaning in life and health care use: findings from a nationally representative study of older adults in Germany. BMC Geriatr.

[15] Guan M. Measuring the effects of socioeconomic factors on mental health among migrants in urban China: a multiple indicators multiple causes model. Int J Ment Heal Syst. 2017;11. 10.1186/s13033-016-0118-y PMC5217273 28070220

[16] Levy-Storms L, Wallace SP (2003). Use of mammography screening among older Samoan women in Los Angeles county: a diffusion network approach. Soc Sci Med.

[17] China NHCo (2016). Chinese health and family planning statistics yearbook. In. Beijing.

[18] Baron RM, Kenny DA (1986). The moderator-mediator variable distinction in social psychological research: conceptual, strategic, and statistical considerations. J Pers Soc Psychol.

[19] Deng Z, Lu Y, Wei KK, Zhang J (2010). Understanding customer satisfaction and loyalty: an empirical study of mobile instant messages in China. Int J Inf Manag.

[20] Vonneilich N, Joeckel K-H, Erbel R, Klein J, Dragano N, Weyers S, Moebus S, Siegrist J, von dem Knesebeck O. Does socioeconomic status affect the association of social relationships and health? A moderator analysis. Int J Equity Health. 2011;10. 10.1186/1475-9276-10-43 PMC3216239 21995609

[21] Zou Q, He X, Li Z, Xu W, Zhang L. The effects of poverty reduction policy on health services utilization among the rural poor: a quasi-experimental study in central and western rural China. Int J Equity Health. 2019;18(1). 10.1186/s12939-019-1099-7 PMC6884802 31783857

[22] Molenaar EA, Van Ameijden EJC, Grobbee DE, Numans ME (2007). Comparison of routine care self-reported and biometrical data on hypertension and diabetes: results of the Utrecht health project. Eur J Pub Health.

[23] Levy D, Larson MG, Vasan RS, Kannel WB, Ho KKL (1996). The progression from hypertension to congestive heart failure. JAMA (Journal of the American Medical Association).

[24] Song H, Zhang D, Chen Z, Wang R, Tang S, Bishwajit G, Chen S, Feng D, Wu T, Wang Y, et al. Utilisation of national community-based blood pressure monitoring service among adult Chinese and its association with hypertension treatment and blood pressure control-a mediation analysis. BMC Geriatr. 2019;19. 10.1186/s12877-019-1176-1 PMC6558874 31182039

[25] Commission SICoCNH (2015). An analysis report of National Health Services Survey in China, 2013. In. Beijing.

[26] Song X, Zou G, Chen W, Han S, Zou X, Ling L (2017). Health service utilisation of rural-to-urban migrants in Guangzhou, China: does employment status matter?. Tropical Med Int Health.

[27] Hesketh T, Jun YX, Lu L, Mei WH (2008). Health status and access to health care of migrant workers in China. Public Health Rep.

[28] Dunn JR, Dyck I (2000). Social determinants of health in Canada's immigrant population: results from the National Population Health Survey. Soc Sci Med.

[29] Glei DA, Goldman N (2000). Understanding ethnic variation in pregnancy-related care in rural Guatemala. Ethnicity & Health.

[30] Alemi Q, Stempel C, Koga PM, Smith V, Danis D, Baek K, Montgomery S (2017). Determinants of Health Care Services Utilization among First Generation Afghan Migrants in Istanbul. Int J Environ Res Public Health.

[31] Chen H, Chi I, Liu R (2019). Hospital utilization among Chinese older adults: patterns and predictors. J Aging and Health.

[32] Li L, Fu H (2017). China's health care system reform: Progress and prospects. Int J Health Plann Manag.

[33] Chen C-C, Lin Y-J, Lin Y-T (2013). Awareness and utilization of preventive care services among the elderly under National Health Insurance. Int J Health Care Finance Economics.

[34] Wang Q. Health of the Elderly Migration Population in China: Benefit from Individual and Local Socioeconomic Status? Int J Environmental Res Public Health. 2017;14(4). 10.3390/ijerph14040370 PMC5409571 28368314

[35] Lu C-H, Luo Z-C, Wang J-J, Zhong J-H, Wang P-X (2015). Health-related quality of life and health service utilization in Chinese rural-to-urban migrant workers. Int J Environ Res Public Health.

[36] Liang Z, Li Z, Ma Z (2014). Changing patterns of the floating population in China, 2000-2010. Popul Dev Rev.

[37] Hone T, Rasella D, Barreto ML, Majeed A, Millett C. Association between expansion of primary healthcare and racial inequalities in mortality amenable to primary care in Brazil: A national longitudinal analysis. PLoS Med. 2017;14(5). 10.1371/journal.pmed.1002306 PMC5448733 28557989

[38] George A, Young M, Bang A, Chan KY, Rudan I, Victora CG, Chopra M, Rubens C. S GEGCB: Setting Implementation Research Priorities to Reduce Preterm Births and Stillbirths at the Community Level. PLoS Med. 2011;8(1). 10.1371/journal.pmed.1000380 PMC3014929 21245907

[39] Li C, Jiang S, Zhang X (2019). Intergenerational relationship, family social support, and depression among Chinese elderly: a structural equation modeling analysis. J Affect Disord.

[40] De Luca G, Ponzo M, Rodriguez Andres A (2013). Health care utilization by immigrants in Italy. International Journal of Health Care Finance & Economics.

